# Biomaterials in tissue repair and regeneration: key insights from extracellular matrix biology

**DOI:** 10.3389/fmedt.2025.1565810

**Published:** 2025-08-15

**Authors:** Sakhavat Abolhasani, Yasin Ahmadi, Yavar Rostami, Erfan Baravar, Davood Fattahi

**Affiliations:** ^1^ Stem Cell Research Center, Tabriz University of Medical Sciences, Tabriz, Iran; ^2^ Student Research Committee, Sarab Faculty of Medical Sciences, Sarab, East Azerbaijan, Iran; ^3^ Department of Medical Laboratory Science, Komar University of Science and Technology, Sulaymaniyah, Kurdistan Region, Iraq; ^4^ School of Pharmacy and Biomolecular Sciences, Liverpool John Moores University, Liverpool, United Kingdom

**Keywords:** extracellular matrix, tissue engineering, regenerative medicine, biomaterials, decellularization, 3D bioprinting, integrin signaling, wound healing

## Abstract

The extracellular matrix (ECM) serves as a dynamic biological framework that orchestrates cellular behavior through biomechanical and biochemical cues, playing a pivotal role in tissue homeostasis and repair. Despite significant advancements in biomaterial design, current regenerative strategies often fail to fully replicate the ECM's complexity, leading to suboptimal healing outcomes. This review comprehensively examines ECM biology and its application in biomaterial engineering, highlighting structural-functional relationships, integrin-mediated signaling, and ECM remodeling mechanisms in wound healing. We analyze diverse biomaterial classes—including ECM-based scaffolds, synthetic polymers, natural biomaterials, bioceramics, and composites—focusing on their design principles, fabrication techniques, degradation profiles, and clinical applications. Key challenges such as immunogenicity, vascularization, mechanical mismatch, and regulatory hurdles are critically evaluated. Innovations in decellularization, biofunctionalization, and advanced manufacturing (e.g., 3D bioprinting, electrospinning) are discussed as promising avenues to enhance biomimicry and therapeutic efficacy. Furthermore, we explore clinically approved ECM-derived products and underscore the need for standardized protocols to bridge translational gaps. By integrating emerging research with clinical perspectives, this review provides a roadmap for developing next-generation ECM-inspired biomaterials that address unmet needs in regenerative medicine, emphasizing interdisciplinary collaboration to optimize safety, functionality, and patient outcomes.

## 1 Introduction

The extracellular matrix (ECM) represents a highly sophisticated biological framework that transcends its conventional role as a passive structural scaffold (1, 2). Comprising a dynamic network of proteins, glycosaminoglycans, and signaling molecules, the ECM actively orchestrates fundamental cellular processes—including adhesion, migration, proliferation, and differentiation—through integrated biomechanical and biochemical cues (3–5). This regulatory capacity arises from its tissue-specific composition and architecture, making it indispensable for physiological homeostasis and a critical blueprint for biomaterial design in regenerative medicine (6, 7). The rising global burden of chronic wounds, degenerative diseases, and organ failure has intensified the demand for advanced therapeutic strategies that address the limitations of conventional treatments (8). While current biomaterials often fail to recapitulate the ECM's dynamic reciprocity with cells (9)—leading to suboptimal outcomes such as fibrosis or functional deficits (10)—recent innovations have yielded ECM-inspired platforms with enhanced biomimicry (11). These span natural polymers (e.g., collagen, hyaluronic acid) (12), synthetic systems (e.g., PLGA, PEG) (13), and hybrid constructs, each offering tunable biocompatibility, mechanics, and bioactivity (14). Concurrent advances in fabrication technologies—such as 3D bioprinting (15), electrospinning (16), and microfluidic patterning (17)—now enable precise replication of the ECM's hierarchical architecture, further augmented by biofunctionalization with peptides (18), glycosaminoglycan mimetics (19), and nanostructured coatings (20). Stimuli-responsive biomaterials exemplify particular promise, dynamically interfacing with host tissues through controlled growth factor release (21) or adaptive mechanical properties (22).

**Figure 1:**
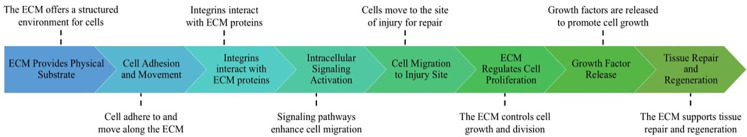
The ECM supports cell migration and proliferation in tissue repair by creating a structured environment and interacting with integrin receptors, while growth factors promote cell proliferation and influence the cell cycle, enhancing tissue regeneration.

Central to the ECM's therapeutic relevance is its dual role in tissue repair: as a structural scaffold and a signaling hub. Following injury, it directs hemostasis, inflammation, proliferation, and remodeling by spatially coordinating cellular responses (23). Key components like fibronectin and collagen engage integrin receptors (24, 25), activating FAK/ERK pathways to drive migration (26, 27) while sequestered growth factors (e.g., TGF-β, PDGF) are released to modulate proliferation (26, 28–32). This synchronized regulation of adhesion, motility, and cell cycle progression creates an optimized microenvironment for regeneration (33–36) (Figure 1).

Despite these advances, critical translational challenges persist. Gaps remain in understanding how engineered ECM analogs influence regenerative outcomes (37), particularly in mimicking dynamic remodeling (38). Immune responses (39), mechanical mismatches (40), and inadequate vascularization (41) further complicate clinical implementation. This review systematically examines ECM biology and its biomaterial applications, analyzing: (i) structure-function relationships governing cell fate; (ii) molecular signaling mechanisms; (iii) comparative advantages of biomaterial classes; and (iv) strategies to overcome immunological, manufacturing, and regulatory barriers. By integrating these perspectives, we aim to accelerate the development of ECM-inspired therapies that bridge the gap between bench innovation and clinical impact.

## 2 Integrin-mediated signaling in tissue repair and regeneration

**Figure 2:**
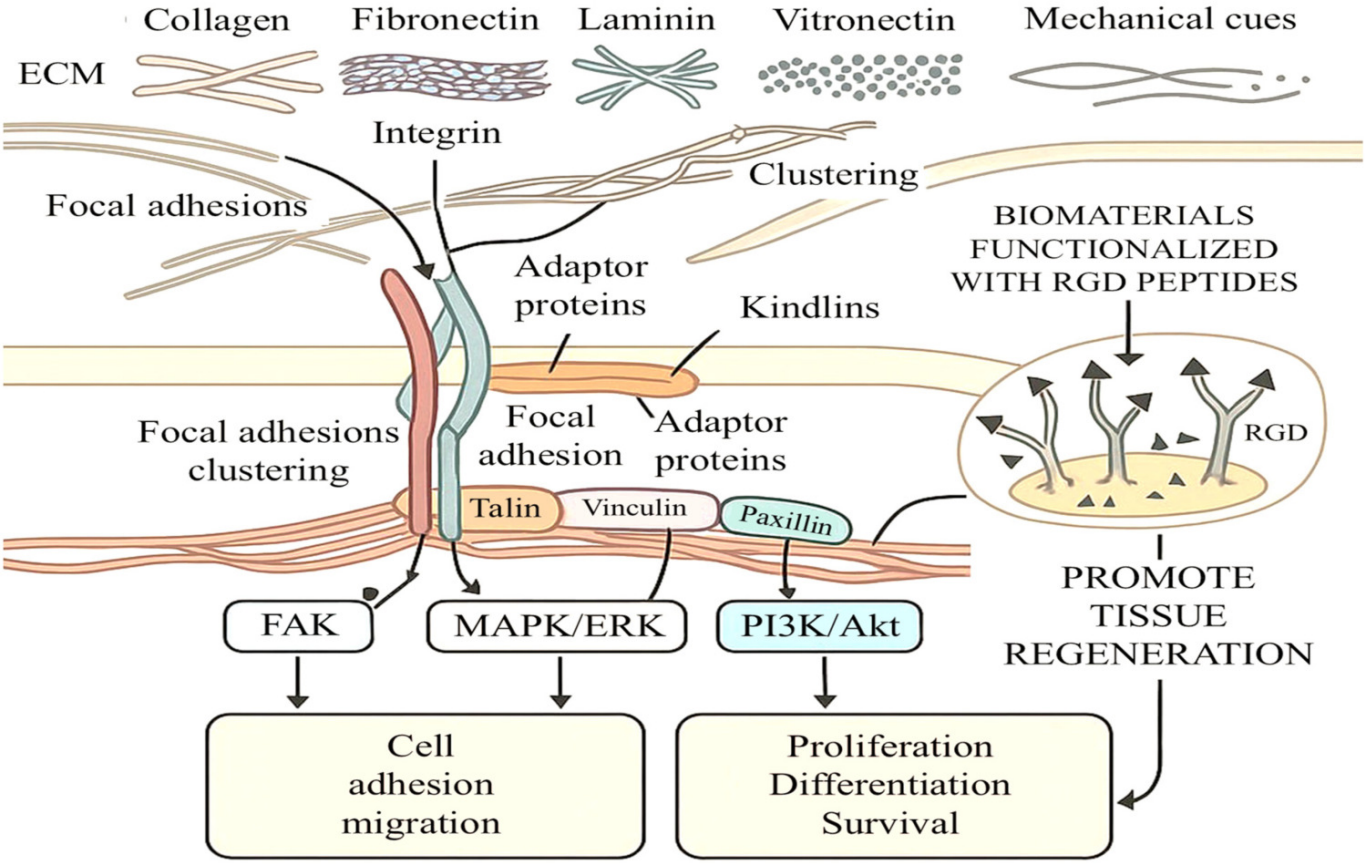
Integrin-mediated signaling in tissue repair and regeneration: integrins, transmembrane receptors binding to ECM components, undergo conformational changes and form focal adhesions. This activates FAK, MAPK/ERK, and PI3K/Akt pathways, regulating cell adhesion, migration, proliferation, and survival. ECM mechanical properties and bioengineered materials modulate integrin signaling to enhance tissue regeneration.

Integrins serve as fundamental mediators of bidirectional communication between cells and their ECM microenvironment, playing indispensable roles in tissue repair and regeneration. These transmembrane receptors, composed of α and β subunits, recognize specific ECM components including collagen, fibronectin, and laminin, thereby orchestrating essential cellular processes such as adhesion, migration, proliferation, and survival (Figure 2) (42). The dynamic interplay between integrins and their ECM ligands forms the molecular foundation for tissue regeneration, with distinct subunit combinations conferring specificity to these critical interactions (43).

The activation of integrin signaling initiates with ECM ligand binding, which induces conformational changes that promote receptor clustering and the assembly of focal adhesion complexes (44). These specialized structures serve as mechanical and biochemical signaling hubs, recruiting adaptor proteins including talin, vinculin, and paxillin to bridge the connection between integrins and the actin cytoskeleton. The formation of focal adhesions triggers the activation of multiple downstream signaling pathways that collectively coordinate the cellular response to tissue injury (45).

Central to this signaling network is the focal adhesion kinase (FAK) pathway, which, upon activation at Tyr397, recruits Src family kinases to regulate cytoskeletal dynamics and promote cell migration (46, 47). Parallel MAPK/ERK pathway activation regulates gene expression for proliferation and differentiation, while the PI3K/Akt pathway promotes cell survival in stressful, injured tissue microenvironments (48–50). These interconnected pathways function synergistically to ensure appropriate cellular responses during the repair process (51).

The mechanical properties of the ECM exert a profound influence on integrin signaling dynamics. Substrate stiffness, topography, and ligand density collectively modulate the spatial organization and activation state of integrin clusters (44, 52). This mechanosensitive regulation of integrin function has inspired innovative biomaterial design strategies aimed at recapitulating key aspects of native ECM signaling. Engineered matrices incorporating RGD peptide sequences demonstrate enhanced capacity to promote cell adhesion and migration through selective engagement of αvβ3 and α5β1 integrins (53, 54).

Recent advances in regenerative medicine have yielded sophisticated biomaterial systems capable of dynamic interaction with integrin receptors. Mineralized scaffolds functionalized with integrin-binding peptides promote osteogenic differentiation of mesenchymal stem cells, while cardiac-specific matrices improve tissue integration following myocardial injury (55, 56). Particularly promising are stimuli-responsive platforms that adapt their presentation of integrin ligands in response to local mechanical or biochemical cues, thereby providing temporal control over regenerative processes (57).

Deciphering integrin signaling pathways enables advanced biomaterial design for regenerative medicine. Targeted modulation of these pathways enhances cellular responses, tissue integration, and therapeutic efficacy while reducing side effects. Key innovations involve nanostructured materials for enhanced integrin clustering, multi-ligand systems for simultaneous integrin engagement, and responsive biomaterials that adapt to physiological cues. These approaches advance regenerative therapies beyond structural mimicry to active biological control, enabling complex tissue restoration (58, 59).

## 3 Dynamic ECM remodeling in wound healing

**Figure 3:**
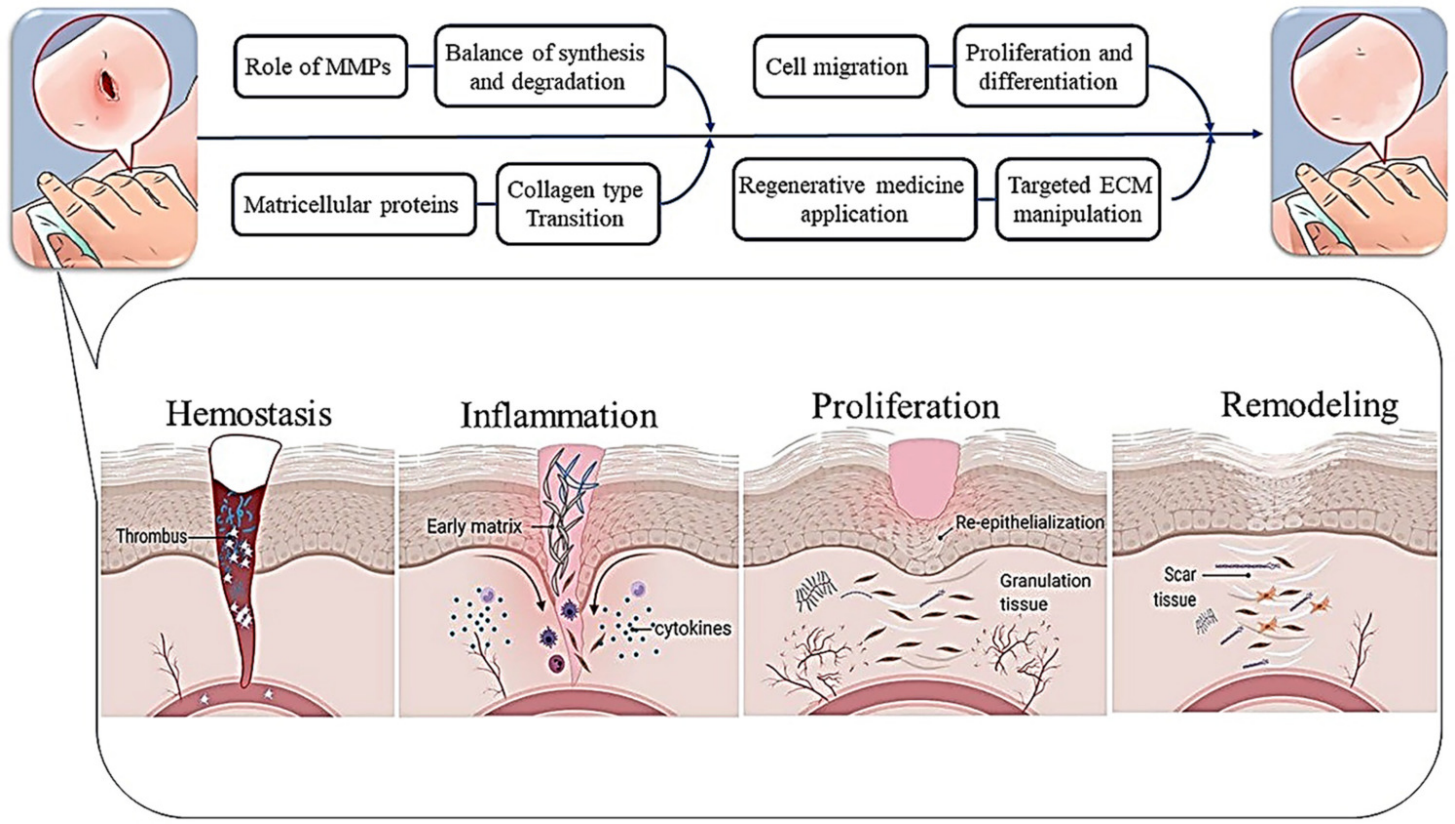
The dynamic process of ECM remodeling during wound healing, highlighting its key phases and components. Following an injury, a fibrin-rich provisional matrix is quickly established, providing structural support and facilitating cellular infiltration to initiate healing. As healing progresses, MMPs degrade this provisional matrix, enabling fibroblast migration and the synthesis of new ECM components. A significant transition occurs from type III to type I collagen deposition, enhancing tissue strength. The remodeling phase is characterized by increased matricellular proteins like fibronectin and tenascin-C, which influence cell-ECM interactions. Maintaining a balance between ECM synthesis and degradation is crucial to prevent complications such as hypertrophic scars.

ECM remodeling is a dynamic, tightly regulated process essential for wound healing, involving degradation of the provisional matrix and deposition of new ECM components critical for tissue restoration (60, 61). Shortly after injury, a fibrin-rich provisional matrix forms, offering structural support and enabling cellular infiltration that initiates repair (62, 63). This matrix also modulates the inflammatory response by recruiting fibroblasts and endothelial cells (64, 65). Matrix metalloproteinases (MMPs) become pivotal during the remodeling phase by degrading the provisional matrix and facilitating fibroblast migration and ECM synthesis (66, 67). MMPs ensure a balanced transition from matrix degradation to new ECM formation, which is essential for effective healing (68). A hallmark of this phase is the replacement of type III collagen with type I collagen, enhancing tissue tensile strength and restoring structural integrity (69–71); see Figure 3. Moreover, remodeling involves upregulation of matricellular proteins like fibronectin and tenascin-C, which modulate cell-ECM interactions and influence cell behavior, including adhesion, migration, and differentiation (72, 73). Precise regulation of ECM turnover is crucial; dysregulation can lead to pathological scarring, such as hypertrophic scars or keloids (74, 75). Overall, ECM remodeling supports both early repair and later tissue normalization through coordinated synthesis and degradation (61).

## 4 ECM-inspired biomaterials

ECM-inspired biomaterials have emerged as a significant advancement in the field of tissue engineering, presenting promising approaches for the repair and regeneration of damaged tissues (76). These biomaterials are engineered to replicate both the structural and biochemical characteristics of the natural ECM, providing an optimal environment conducive to cellular activities critical for healing (77). The inherent properties of the ECM are being investigated in efforts to develop scaffolds that promote cell attachment and proliferation while also enhancing the intricate processes of tissue repair and remodeling (78). The creation of these biomaterials is underpinned by a comprehensive understanding of the ECM's functions in physiological processes, thus positioning them as essential tools within the realm of regenerative medicine (79).

### 4.1 Design principles and material selection of ECM-inspired biomaterials

The design principles underlying ECM-inspired biomaterials focus on the precise replication of the architecture, composition, and mechanical properties characteristic of the native ECM (80). Critical factors in material selection involve the origin of ECM components, the techniques employed for decellularization, and the integration of bioactive molecules enhancing cell signaling and facilitating tissue integration (81). Commonly utilized materials, such as collagen, gelatin, fibrin, and hyaluronic acid, are favored not only for their inherent biocompatibility but also for their capacity to support fundamental cellular processes, including adhesion, migration, and proliferation (Table 1) (82). Among these, collagen is particularly prominent due to its abundance in mammalian ECM and its ability to impart tensile strength and structural integrity to engineered tissues (83).

**Table 1:** The diversity of ECM-inspired biomaterials, their compositions, architectures, mechanical properties, and applications.

Type of ECM- Inspired Biomaterial	Composition	Architecture	Mechanical Properties	Applications
Decellularized Tissues	Natural ECM proteins (collagen, elastin)	Retains native tissue structure	Variable stiffness, mimics native tissue	Tissue engineering, regenerative medicine (90)
		3D structure reflects original organ architecture		Organ transplantation, vascular grafts (90)
Synthetic Hydrogels	Polyethylene glycol (PEG), hyaluronic acid	3D porous networks	Tunable mechanical properties, adjustable viscosity	Drug delivery, cell culture, wound healing (91)
		Hydrophilic networks facilitate nutrient transport		Soft tissue repair, tissue engineering (91)
Self-Assembled Peptide Scaffolds	Peptides designed to mimic ECM components	Nanofibrous structures	Adjustable stiffness and elasticity	Tissue repair, 3D cell culture (92)
		Self-assembly can be tuned for specific applications		Bone regeneration, guided tissue regeneration (92)
Composite Biomaterials	Combination of natural and synthetic polymers	Layered or hybrid structures	Enhanced mechanical strength and flexibility	Bone regeneration, soft tissue repair (93)
	Often include bioactive glass or ceramics	Interconnected porosity promotes cell infiltration		Cartilage repair, orthopedic applications (93)
Electrospun Fibers	Collagen, gelatin, or synthetic polymers	Fibrous mats with high surface area	High tensile strength and flexibility	Nerve regeneration, wound healing (94)
	Can include blended polymers for enhanced properties	Mimics the structure of native ECM		Drug delivery vehicles, tissue scaffolding (94)
Bio-inks for 3D Printing	Natural polymers (alginate, gelatin)	Customizable structures based on design	Varies based on formulation	Organ-on-chip models, personalized tissue scaffolds (95)
	Often combined with cells for bioprinting	Controlled architecture enables multi-layering		Custom prosthetics, tissue mimicry (95)
Nanoparticle-Integrated Biomaterials	Biodegradable polymers with nanoparticles	3D or 2D structures	Variable stiffness due to integration of nanoparticles	Drug delivery, cancer therapy (96)
	Can include gold, silver, or silica particles	Enhanced mechanical and bioactivity properties		Imaging and diagnosis tools (96)
Conductive Biomaterials	Polymers with conductive properties (e.g., PEDOT: PSS)	Scaffold frameworks for cell adhesion	Electrical conductivity enhances cellular responses	Neural tissue engineering, cardiac tissue repair (97)
	Often incorporated with growth factors	Can be made 3D-printed or electrospun		Bioelectronics, sensors within the body (97)
Responsive Hydrogels	Stimuli-responsive polymers (e.g., pH or temperature-sensitive)	Swell and shrink upon stimulus	Mechanical properties change with environmental conditions	Drug delivery systems, smart wound dressings (98)
	Often includes additives for responsiveness	Dynamic architecture can enhance function		Diagnostic applications, environmental sensing (98)

The methods of decellularization are crucial in the synthesis of ECM-based biomaterials, as these techniques strive to eliminate cellular components while preserving the structural integrity and bioactive properties of the native matrix (84). Various techniques, including chemical treatments, enzymatic digestion, and physical approaches such as freeze-thaw cycles, can be effectively utilized to achieve successful decellularization (85). The selection of a specific decellularization method significantly influences the resulting material's properties, affecting mechanical strength, porosity, and degradation kinetics. Furthermore, subsequent processing steps—such as crosslinking and sterilization—are essential to enhance the stability, durability, and overall functionality of these biomaterials (86).

In addition to selection and processing methods, the incorporation of spatial patterning techniques further enhances the functionality of ECM-inspired biomaterials. These techniques facilitate the design of scaffolds with specific microarchitectures that accurately replicate the native tissue environment (87). Techniques such as photolithography and electrospinning allow for precise manipulation of ECM component distribution at the micro- and nanoscale (88, 89). This spatial control is crucial for promoting proper organization and alignment of cells within the scaffold, ultimately improving tissue integration and enhancing the overall functionality of the engineered tissue constructs.

## 5 Biomaterial classes, properties, fabrication techniques, and degradation profiles in tissue repair and regeneration

Biomaterials used in tissue engineering are broadly classified into five main categories based on their composition, physicochemical properties, and biological functions: (1) ECM-based biomaterials, (2) synthetic polymers, (3) natural biomaterials, (4) bioceramics, and (5) composites. Each class exhibits unique physical, chemical, and biological characteristics that determine its suitability for specific regenerative applications (99).

A pivotal factor influencing biomaterial efficacy is their degradation profile. Effective biomaterials degrade at rates synchronized with tissue formation, maintaining scaffold integrity during healing. Additionally, their degradation products must be non-toxic, readily cleared, and supportive of the regenerative environment, ensuring optimal scaffold performance without adverse effects (100).

A comprehensive understanding of these materials' properties alongside their fabrication methods is essential for designing scaffolds and devices that optimize cell–material interactions, mechanical stability, and degradation kinetics, all critical to successful tissue regeneration. The subsequent sections detail the composition, functionalities, and manufacturing techniques associated with these biomaterial classes (Table 2).

**Table 2:** Summary of physical/chemical properties and fabrication techniques of biomaterial classes.

Biomaterial Class	Key Properties	Common Fabrication Techniques
ECM-based	Native composition, high bioactivity, porous	Decellularization, lyophilization, 3D bioprinting (90)
Synthetic polymers	Tunable, reproducible, controlled degradation	Electrospinning, 3D printing, solvent casting (101)
Natural biomaterials	Biocompatible, biodegradable, variable strength	Gelation, crosslinking, freeze-drying (102)
Bioceramics	Osteoconductive, strong, brittle	Sintering, sol-gel, 3D printing (103)
Composites	Synergistic, customizable, multifunctional	Co-electrospinning, particulate leaching (104)

### 5.1 ECM-based biomaterials

ECM-based biomaterials comprise decellularized tissue matrices, ECM-derived hydrogels, and self-assembled scaffolds engineered to replicate the biochemical and biophysical properties of the native ECM. These materials are inherently bioactive, containing crucial biological cues such as growth factors, glycosaminoglycans, and adhesive motifs (e.g., RGD peptides), which regulate cellular behaviors including adhesion, migration, proliferation, and differentiation via integrin-mediated and other signaling pathways (105). Structurally, ECM scaffolds exhibit a porous, fibrillar architecture that promotes cell infiltration and nutrient diffusion, essential for effective tissue regeneration. However, their mechanical properties vary with tissue origin and decellularization methods, often necessitating reinforcement for load-bearing applications (106, 107).

Fabrication approaches include physical, chemical, or enzymatic decellularization, enzymatic digestion to produce hydrogels, and lyophilization for porous constructs. Advanced techniques such as electrospinning yield nanofibrous matrices mimicking native microarchitecture, while 3D bioprinting allows precise spatial deposition of ECM components, enabling complex, organ-specific scaffold fabrication with enhanced reproducibility (101, 108). Innovations in decellularization focus on preserving ECM ultrastructure and bioactivity to support stem cell incorporation and growth factor delivery, with tissue-specific hydrogels demonstrating potential for minimally invasive therapies due to their injectability and remodeling capacity (81, 109). Clinically, ECM-based scaffolds are applied in cardiac repair, vascular grafts, wound healing, and ligament reconstruction, with several products achieving regulatory approval or undergoing clinical trials, underscoring their translational relevance (110).

ECM biomaterials primarily degrade *via* enzymatic pathways involving MMPs, collagenases, and other proteases targeting collagen, elastin, and glycosaminoglycans. This tightly regulated remodeling mirrors natural tissue turnover and wound healing processes (111). Importantly, degradation by-products are generally biocompatible and may actively enhance regeneration by releasing bioactive peptides that stimulate cell migration, proliferation, angiogenesis, and matrix synthesis. For example, collagen-derived peptides can serve as chemotactic factors to guide cell infiltration. Nonetheless, the degradation rate must be carefully balanced: excessively rapid breakdown can undermine scaffold integrity and tissue formation, whereas overly slow degradation may hinder tissue remodeling and integration (112).

### 5.2 Synthetic polymers

Synthetic polymers, including polylactic acid (PLA), polyglycolic acid (PGA), poly lactic-co-glycolic acid (PLGA), and polycaprolactone (PCL), have become widely utilized scaffolding materials in regenerative medicine due to their tunable mechanical properties, controlled degradation rates, and ease of processing (113, 114). These polymers offer high reproducibility, scalability, and well-defined chemical structures, enabling precise modulation of key physical and chemical characteristics such as hydrophilicity, stiffness, and degradation kinetics. Such versatility renders them suitable for diverse tissue engineering applications spanning cartilage, bone, nerve, and soft tissue regeneration (115).

To augment bioactivity and enhance functional integration, extensive research has focused on modifying polymer architecture through variations in copolymer ratios and molecular weights, and implementing surface engineering strategies such as plasma treatment, peptide grafting, and biomimetic coatings. These approaches mimic biological cues or facilitate the incorporation of growth factors, thereby promoting cell adhesion, proliferation, and differentiation (116). Fabrication techniques, including solvent casting, melt extrusion, and electrospinning, allow the generation of films, fibers, and nanofibrous matrices that replicate ECM features, while 3D printing enables the creation of anatomically precise scaffolds with customizable porosity and spatial patterning (101, 117).

Degradation of synthetic polymers primarily occurs via hydrolysis of ester bonds in the polymer backbone, with the degradation rate modulated by factors such as copolymer composition, molecular weight, crystallinity, and scaffold geometry. This predictable and tunable degradation is advantageous for synchronizing scaffold resorption with tissue formation. However, hydrolysis generates acidic by-products—lactic and glycolic acids—that can lower local pH, potentially induce inflammation or cytotoxic effects if not adequately buffer by surrounding tissues or scaffold design (118). Therefore, balancing degradation kinetics with biocompatibility through careful polymer selection and scaffold architecture is essential to maintain a conducive microenvironment for cell viability and tissue regeneration.

### 5.3 Natural biomaterials

Natural biomaterials—such as collagen, gelatin, chitosan, alginate, and hyaluronic acid—are extensively utilized in tissue engineering owing to their inherent biocompatibility, biodegradability, and capacity to closely mimic native ECM components (119, 120). These polymers inherently contain bioactive motifs that facilitate cellular adhesion, proliferation, and differentiation, making them particularly suitable for regenerative applications across cartilage, skin, nerve, and soft tissues (121).

Despite these biological advantages, natural polymers often exhibit mechanical limitations, including relatively low stiffness and significant batch-to-batch variability, which can compromise reproducibility and long-term scaffold stability (122). To overcome these constraints, chemical and physical modification strategies have been applied to enhance mechanical properties and tailor biofunctional characteristics. Fabrication methods commonly employed include ionic or covalent crosslinking to induce gelation, freeze-drying to create porous scaffolds, electrospinning to produce fibrous structures, and photo-crosslinking to fine-tune mechanical stiffness and degradation kinetics in response to cellular needs (123, 124). These techniques enable the formation of versatile scaffold architectures, such as hydrogels, sponges, and films, which are frequently blended with synthetic polymers to improve mechanical strength and control degradation profiles (125).

Recent advances highlight the potential of crosslinked collagen hydrogels in enhancing mechanical stability and directing stem cell differentiation within cartilage and skin regeneration models. Gelatin meth acryloyl (GelMA) has emerged as a prominent biomaterial due to its tunable rheological properties and compatibility with 3D bioprinting technologies, facilitating the fabrication of vascularized tissue constructs (126, 127). Similarly, chitosan derivatives with improved solubility and functionalization have demonstrated efficacy in nerve and cartilage tissue engineering, while RGD-modified alginate scaffolds have shown enhanced mesenchymal stem cell adhesion and osteochondral differentiation. Hyaluronic acid-based hydrogels with engineered stiffness have been successfully applied in wound healing, synovial joint repair, and neural regeneration (128, 129).

In terms of degradation, natural polymers typically undergo enzymatic breakdown or hydrolysis at rates generally faster than synthetic polymers. Enzymes such as collagenases and lysozymes secreted by cells mediate scaffold resorption. The resulting degradation products—oligosaccharides, peptides, and amino acids—are largely non-toxic and well-integrated within cellular metabolic pathways, often facilitating tissue remodeling and integration. However, excessively rapid degradation can undermine scaffold mechanical integrity prematurely, potentially compromising support during critical phases of tissue regeneration.

### 5.4 Bioceramics

Bioceramics, including hydroxyapatite (HA), tricalcium phosphate (TCP), and bioactive glasses, are pivotal materials in bone and dental tissue engineering due to their remarkable osteoconductivity, chemical stability, and high compressive strength, which collectively confer suitability for load-bearing applications (130, 131). These ceramics facilitate direct bonding with native bone tissue, providing essential structural support during the regenerative process. However, their inherent brittleness and limited mechanical flexibility constrain their applicability in soft tissue engineering, while their relatively slow degradation rates may impede complete tissue remodeling unless carefully tailored.

To enhance their bioactivity and regenerative potential, extensive research has focused on nanostructuring bioceramics to increase surface area, thereby promoting improved cellular adhesion, proliferation, and osteogenic differentiation (132). Ion-doping approaches—such as substitution with strontium (Sr²^+^) or silicon (Si⁴^+^) ions—have been demonstrated to stimulate osteogenesis, angiogenesis, and exhibit anti-resorptive properties *in vivo*, further augmenting the therapeutic efficacy of these materials (133).

Composite scaffolds that integrate bioceramics with biodegradable polymers, growth factors, or stem cells exhibit synergistic effects, accelerating bone healing and enabling controlled resorption, particularly advantageous for large or complex osseous defects (134). Advances in additive manufacturing, including 3D printing technologies, have facilitated the fabrication of patient-specific ceramic implants with precisely engineered porosity and mechanical properties. These innovations optimize vascular infiltration and promote robust integration with host tissue (135, 136).

Clinically, bioceramics are widely employed in dental implants, craniofacial reconstruction, and orthopedic devices, where they have demonstrated long-term biocompatibility, mechanical durability, and effective osseointegration (137). Fabrication techniques such as sintering, sol-gel processing, and surface modifications—including micro topographical roughening and bioactive coatings—are employed to enhance cellular attachment, vascularization, and implant stability, thereby improving functional outcomes.

Bioceramics predominantly undergo degradation via slow dissolution in physiological fluids and active cellular resorption by osteoclast-like cells. The rate of degradation is influenced by parameters including material porosity, crystallinity, and surface chemistry (138).

The gradual resorption profile of bioceramics ensures sustained mechanical support during critical phases of bone regeneration. As degradation proceeds, released calcium and phosphate ions contribute to new mineralized tissue formation. Nevertheless, incomplete resorption or accumulation of ceramic debris may impede full tissue remodeling or elicit inflammatory responses if degradation kinetics and scaffold design are not properly controlled.

### 5.5 Composites

Composite biomaterials represent a strategic amalgamation of diverse material classes—such as synthetic polymers, bioceramics, and biological macromolecules—engineered to synergistically enhance mechanical strength, bioactivity, and biodegradability to address the complex demands of tissue regeneration (139). By combining the advantageous properties of each component, these hybrid systems overcome the intrinsic limitations of individual materials, enabling the development of scaffolds with precisely tailored physicochemical and biological characteristics.

For instance, polymer-ceramic composites like PCL blended with HA have demonstrated improved osteogenic differentiation and mineral deposition, making them particularly effective in bone tissue engineering (140). The ceramic component contributes essential mechanical reinforcement and osteoconductivity, while the polymer matrix imparts flexibility and facilitates manufacturability. This compositional synergy also allows fine-tuning of degradation kinetics, thereby aligning scaffold resorption with the temporal progression of tissue regeneration.

Advancements in fabrication techniques, especially additive manufacturing and layer-by-layer assembly, have facilitated the production of gradient or stratified scaffolds that closely replicate complex tissue interfaces such as the osteochondral junction (141, 142). The precise spatial control afforded by these technologies supports the recreation of native tissue anisotropy and zonal heterogeneity, which enhances structural integration and promotes functional restoration.

Moreover, the integration of bioactive agents—including bone morphogenetic protein-2 (BMP-2) and vascular endothelial growth factor (VEGF)—within composite scaffolds further stimulates angiogenesis, stem cell recruitment, and tissue repair in critical-sized defects (143, 144). These biofunctional constructs are engineered for controlled, localized, and sustained release of therapeutic factors, optimizing the regenerative microenvironment.

A critical aspect of composite biomaterials is their tailored degradation behavior, which results from the interplay between their constituent components. Degradable polymers typically undergo hydrolytic or enzymatic cleavage, bioceramics degrade via dissolution or cellular resorption, and natural ECM -derived materials are enzymatically broken down. By combining these components, composite scaffolds can be designed to exhibit synergistic or sequential degradation profiles that closely match the tissue healing timeline, ensuring mechanical support is maintained during early regeneration and progressively replaced by newly formed tissue (145).

The biological performance of these composites depends heavily on the compatibility and degradation synchrony of the integrated materials. Properly balanced degradation kinetics prevent premature scaffold fragmentation and promote uniform cellular infiltration and tissue ingrowth, which are essential for effective remodeling. Conversely, mismatched degradation rates or material incompatibility can lead to scaffold instability or heterogeneous regeneration, ultimately compromising functional outcomes.

Composite biomaterials engineered for complex tissue interfaces—such as tendon-to-bone entheses, vascular grafts, and nerve conduits—illustrate the necessity of integrating mechanical robustness with controlled bioactive delivery. Such multifunctional systems meet the dual demands of biomechanical support and localized biological modulation, thereby advancing regenerative therapies toward more predictable and durable clinical success (146, 147).

## 6 Advantages and limitations of biomaterial classes in tissue engineering

The strategic selection of biomaterials for tissue engineering requires careful evaluation of their inherent advantages and limitations across multiple functional parameters. As shown in Table 3, the five principal biomaterial classes—ECM-based, synthetic polymers, natural biomaterials, bioceramics, and composites—each present distinct profiles of biocompatibility, bioactivity, mechanical properties, and manufacturability that dictate their clinical suitability.

**Table 3:** Clinically approved biomaterial-based systems for tissue repair and regeneration.

Product Name	Composition	Clinical Indication	Regulatory Status	Manufacturer
Integra® Dermal Regeneration Template	Bovine collagen + glycosaminoglycan	Skin regeneration, burn treatment	FDA approved, CE marked	Integra LifeSciences (195)
AlloDerm®	Decellularized human dermis	Soft tissue reconstruction, burns	FDA cleared (HCT/P)	LifeCell Corporation (196)
Bio-Gide®	Collagen membrane (porcine)	Guided bone regeneration	CE marked	Geistlich Pharma (197)
Infuse® Bone Graft	Recombinant human BMP-2 + collagen	Spinal fusion, bone defects	FDA approved	Medtronic (198)
GraftJacket®	Acellular human dermis	Chronic wound repair	FDA cleared (HCT/P)	Wright Medical (199)
OsteoCel®	Cellular allograft (bone matrix + MSCs)	Bone regeneration	FDA cleared (HCT/P)	NuVasive (200)
EpiFix®	Dehydrated amniotic membrane	Chronic wound healing	FDA cleared (HCT/P)	MiMedx (201)
Actifuse®	Silicate-substituted calcium phosphate	Bone void filler	FDA cleared, CE marked	Baxter (202)
Chondro-Gide®	Collagen type I/III membrane	Cartilage repair (knee)	CE marked	Geistlich Pharma (203)
Permacol™	Porcine dermal collagen	Soft tissue repair	FDA cleared, CE marked	Medtronic (204)

FDA, U.S. food and drug administration; CE, European conformity; HCT/P, human cells, tissues, and cellular and tissue-based products.

ECM-derived and natural biomaterials excel in biological recognition and cellular signaling but face challenges with immunogenicity (particularly in xenogeneic formulations), batch-to-batch variability, and insufficient mechanical strength for load-bearing applications. Synthetic polymers offer superior reproducibility and tunable properties, though their frequent lack of intrinsic bioactivity and potential for cytotoxic degradation byproducts (e.g., acidic monomers from PLGA hydrolysis) remain significant concerns (148). Bioceramics provide exceptional osteoconductivity and structural stability in bone regeneration, yet their inherent brittleness and processing limitations constrain wider application. Composite systems strategically combine material classes to achieve synergistic performance, though this introduces fabrication complexity and potential interfacial incompatibilities (149).

Beyond class-specific limitations, four fundamental challenges persist across all biomaterial categories (Table 4). First, immune compatibility remains problematic, with ECM-based and natural materials particularly prone to provoking inflammatory responses or rejection through residual xenogeneic antigens. Second, cytotoxic effects may emerge from either degradation byproducts (synthetics) or residual crosslinking agents (natural/ECM materials). Third, physiological integration is frequently compromised by mechanical mismatches or asynchronous degradation kinetics, leading to fibrotic encapsulation or incomplete tissue remodeling. Fourth, long-term safety profiles require further validation, especially regarding late-stage inflammatory responses, mineralization anomalies, or stress-shielding effects from mechanical property disparities.

**Table 4:** Comparative Performance Matrix of Tissue Engineering Biomaterials.

Biomaterial Class	Key Advantages	Primary Limitations	Cross-Cutting Challenges
ECM-based	High bioactivity, native cell signaling, excellent biocompatibility	Immunogenicity, source variability, low mechanical strength	Immune responses (xenogeneic antigens), infection risk, complex sterilization (5)
Synthetic polymers	Highly tunable properties, excellent reproducibility, scalable production	Limited bioactivity, cytotoxic degradation products	Mechanical mismatch, acidic degradation microenvironment (150)
Natural biomaterials	Innate biocompatibility, biodegradability, bioactive motifs	Rapid degradation, immunogenicity, weak mechanics	Batch variability, pathogen risk, crosslinker toxicity (151)
Bioceramics	Superior osteoconductivity, high compressive strength, stability	Brittleness, difficult processing, slow degradation	Stress shielding, poor interfacial integration (152)
Composites	Tailorable properties, synergistic performance, multifunctionality	Complex fabrication, interfacial incompatibility, regulatory hurdles	Phase separation, inconsistent degradation profiles (153)

The risk of infection presents additional translational hurdles, particularly for biological materials that may harbor pathogens or support biofilm formation despite sterilization protocols. Furthermore, the dynamic interplay between scaffold degradation and tissue formation necessitates precise temporal control—overly rapid resorption can compromise structural support, while excessively persistent materials may impede functional tissue maturation.

## 7 Applications of biomaterials in tissue engineering

ECM-inspired biomaterials have been extensively applied in tissue engineering due to their ability to promote repair and regeneration across multiple tissues (154). Notably, in vascular grafts, these materials provide a supportive microenvironment that enhances endothelial cell proliferation and angiogenesis. These biomaterials mimic the mechanical properties of native blood vessels and enhance graft patency. Examples include decellularized vascular tissues and synthetic hydrogels (155–157). Similarly, ECM-derived cardiac patches enhance myocardial repair post-infarction by promoting cell survival, tissue regeneration, and endothelial cell growth, thereby improving angiogenesis. These patches support cardiac cell function and integration, with key examples including decellularized cardiac matrices and elastin-based scaffolds (158, 159). Beyond cardiovascular applications, ECM scaffolds are employed in regenerating skin, bone, cartilage, and nerve tissues (160). Moreover, ECM-based materials have advanced organ-on-a-chip technologies leverage ECM-derived microenvironments to replicate physiological conditions for real-time monitoring of cellular responses, with ECM-derived hydrogels and peptide-composite biomaterials proving particularly effective and enhancing drug testing and disease modeling (161, 162).

### 7.1 Bone tissue regeneration

Bone regeneration research utilizes ECM-derived materials that enhance osteogenic differentiation of stem cells and support bone healing by incorporating bioactive cues and mimicking natural bone structure, such as decellularized bone matrices and HA composites (163). Scaffold design focuses on creating osteoconductive environments that promote osteoinduction through stem cell differentiation and host tissue integration. Bioactive ceramics like biphasic calcium phosphate (BCP) and HA composites enhance osteointegration (164), while emerging 3D-printed scaffolds with bioactive nanoparticles improve *in vivo* osteogenic differentiation (165, 166). Controlled bone formation is achieved via growth factor delivery (e.g., BMP-2, BMP-7) in biodegradable carriers (167). Composite scaffolds combining natural polymers (collagen, chitosan) with ceramics demonstrate superior mechanical and cellular outcomes (168), and surface modifications (nanotopography, biofunctional peptides) further enhance stem cell adhesion and mineralization (169). Key challenges include vascularizing large defects, ensuring long-term growth factor safety, and addressing regulatory and cost barriers for clinical translation.

### 7.2 Cartilage tissue regeneration

Cartilage repair remains challenging due to tissue avascularity and limited self-renewal capacity. Biomaterials designed for cartilage regeneration aim to replicate native mechanical properties while supporting chondrocyte function, with examples including chitosan-based scaffolds and elastin-like polypeptides (170). Hydrogels based on hyaluronic acid, gelatin, or alginate mimic the native ECM to promote chondrogenesis (171), while ECM-derived scaffolds preserve biochemical cues to enhance cell attachment and differentiation (78). Advanced strategies employ composite systems for sustained delivery of growth factors (TGF-β, IGF-1) to stimulate hyaline cartilage formation (172), as well as MSC-laden biomimetic scaffolds and 3D bioprinting to achieve precise spatial architecture (173). Despite progress, key challenges persist in generating durable hyaline cartilage, scaling up manufacturing, and ensuring integration with subchondral bone.

### 7.3 Skin tissue regeneration

Wound healing employs natural and synthetic biomaterials to accelerate closure, reduce scarring, and restore function, especially in chronic wounds and burns. Decellularized dermal matrices and collagen scaffolds improve closure and revascularization (77) and also facilitate keratinocyte migration and re-epithelialization in skin healing (174). Incorporation of bioactive molecules like VEGF enhances angiogenesis (175). Electrospun synthetic polymers (e.g., PCL) support cell infiltration tailored to wound environments (176). Advanced templates incorporating stem cells or exosomes promote superior regeneration (177), while antimicrobial wound dressings mitigate infection risk (178). Balancing bioactivity, degradation, and regulatory approval remains critical.

### 7.4 Nerve tissue regeneration

ECM-inspired scaffolds play a pivotal role in nerve regeneration by providing structural support and essential biochemical signals that enhance neuronal survival and growth while mimicking the natural nerve environment, as demonstrated by decellularized nerve grafts and peptide-based hydrogels (179). Peripheral and central nerve repair strategies focus on axonal guidance, neurogenesis, and functional restoration through multiple approaches. Biodegradable nerve conduits made from PCL, PLA, or ECM-mimetic materials support regeneration (180), with electroactive polymers further enhancing neurotrophic signaling (181). Decellularized nerve scaffolds effectively preserve bioactive cues to facilitate Schwann cell migration (182), while composite conduits incorporating neurotrophic factors (NGF, BDNF) delivered via microspheres or hydrogels show improved regenerative outcomes (183). Advanced 3D bioprinted nerve interfaces with aligned microchannels offer promising solutions for bridging critical nerve gaps (184). Despite these advances, key challenges remain in optimizing scaffold design, ensuring proper vascularization, and navigating regulatory pathways for bioactive conduit approval.

### 7.5 Liver tissue regeneration

Liver regeneration research focuses on replicating architecture, metabolic function, and transplantation alternatives. Decellularized liver matrices retain vascular and biliary structures for hepatocyte recellularization (185). 3D bioprinted liver constructs with multicellular components enhance hepatocyte function (186). Bioartificial liver systems in modular bioreactors serve as bridging therapies for acute failure (187), complemented by microfluidic platforms for viability assessment (188). Major obstacles include vascularization, immune compatibility, and scalable off-the-shelf graft production.

### 7.6 Vascular tissue regeneration

Vascular engineering targets grafts for damaged vessels, from small-diameter grafts to arteries. Decellularized vessels preserve native cues promoting endothelialization (189, 190). Electrospun polymers and elastomers enable compliant, hemocompatible grafts (191). EPC seeding and preconditioning improve patency and thrombogenicity (192). Incorporation of anticoagulants and nitric oxide donors enhances durability (193). Bioprinting vascular networks with hierarchical microchannels advances complex vasculature engineering (194). Challenges include ensuring long-term patency, mechanical compliance, and evolving regulatory frameworks.

### 7.7 Other tissues

Emerging biomaterial applications include muscle (injectable hydrogels), tendon (aligned nanofibers), and lung (decellularized matrices, vascularized bioprinted constructs). Smart biomaterials responsive to mechanical, electrical, or biochemical stimuli and gene delivery systems show promise in enhancing regeneration.

## 8 Clinically approved biomaterial-based systems for tissue repair and regeneration

The transition from laboratory to clinical application represents a critical step in advancing tissue engineering. Successful clinical translation of biomaterials affirms their safety, efficacy, and therapeutic potential. Over recent decades, multiple biomaterial-based systems have gained regulatory approval for various tissue regeneration applications. Table 3 summarizes approved biomaterial products, highlighting their diverse compositions, clinical uses, and regulatory statuses.

The progression of these biomaterials into clinical practice reflects significant advancements in biomaterial science, manufacturing scalability, and understanding of tissue-specific regenerative cues. Notably, many approved systems incorporate natural components that mimic native ECM, fostering integration and functional tissue regeneration.

Moreover, the regulatory landscape reveals a growing acceptance of advanced biological products, such as decellularized tissues (AlloDerm®) and cellular allografts (OsteoCel®), which offer superior bioactivity. The inclusion of growth factors (e.g., recombinant BMP-2 in Infuse®) demonstrates the increasing reliance on bioactive molecules to stimulate regenerative processes.

## 9 Regulatory considerations for ECM-based biomaterials

Regulatory considerations for ECM-based biomaterials involve a variety of factors, including the selection of source tissues, decellularization techniques, and subsequent processing methods (205). The origin of the tissue—whether allogeneic or xenogeneic—can significantly influence the immunogenicity and biocompatibility of the resultant product (206). Regulatory authorities mandate a thorough evaluation of these materials to ensure compliance with stringent safety standards, which includes assessments of potential inflammatory responses and long-term biocompatibility (207). For example, effective decellularization is essential for removing cellular components that could trigger an immune response (208); however, existing guidelines lack standardized criteria for evaluating the sufficiency of decellularization processes (209). This lack of standardization can result in inconsistencies in clinical outcomes, as some commercially available ECM scaffolds may not fully adhere to established criteria, yet still show favorable results in practice (210).

Furthermore, the manufacturing process of ECM-based biomaterials is complex and typically involves multiple steps that can modify their physical and biochemical characteristics (211). These alterations can affect cellular behavior and tissue integration following implantation. Consequently, regulatory agencies must evaluate not only the final product but also the entire manufacturing process when assessing ECM-based biomaterials (212). This comprehensive approach is vital to ensure that these materials achieve their intended therapeutic objectives while minimizing associated risks. As research in this field progresses, the establishment of clear guidelines and standardized protocols will be essential for streamlining the regulatory approval process for ECM-inspired biomaterials.

## 10 Innovations in ECM biomaterials research

Advancements in ECM biomaterials research are crucial for overcoming current challenges and enhancing their functionality in tissue engineering applications (213). Recent developments have concentrated on enhancing the mechanical properties and bioactivity of ECM-derived materials through various strategies (214). Researchers are exploring innovative decellularization methods that preserve the structural integrity and biological functionality of ECM components while effectively removing cellular debris (84). Furthermore, incorporating bioactive molecules, such as growth factors or peptides, into ECM scaffolds shows potential in promoting specific cellular responses that aid tissue regeneration (5).

Another promising area of research involves the creation of hybrid biomaterials that combine ECM components with synthetic polymers or other materials, resulting in scaffolds with customized properties (14). These hybrid systems capitalize on the benefits of both natural and synthetic materials, providing enhanced mechanical strength while preserving critical bioactive characteristics necessary for cell signaling and tissue integration (14). Furthermore, Advancements in fabrication techniques, such as 3D bioprinting and electrospinning, allow for the creation of complex scaffold architectures that better replicate native tissue environments, enhancing cellular behavior and improving healing outcomes (88).

## 11 Translational gaps in ECM-inspired biomaterials

Despite significant advances in ECM-inspired biomaterials for tissue repair and regeneration, several scientific and translational gaps persist. Addressing these challenges is essential for the successful clinical translation and optimization of biomaterial-based therapies (Table 5).

**Table 5:** Key gaps and challenges.

Gap/Challenge	Concrete Example(s)	Ongoing Efforts/Recent Advances
Immunogenicity	Decellularized ECM from animal sources can trigger immune responses and fibrosis in host tissue.	Improved decellularization protocols; use of human-derived ECM (215).
Integration with Host Tissue	Synthetic scaffolds often fail to integrate, leading to encapsulation or poor vascularization.	Surface modification with bioactive peptides; co-delivery of angiogenic factors (216).
Vascularization	Large engineered constructs lack sufficient blood vessel ingrowth, limiting nutrient diffusion.	Incorporation of pro-angiogenic cues; pre-vascularized scaffolds (217).
Batch-to-Batch Variability	ECM-derived materials show inconsistent mechanical and biochemical properties due to source variation.	Standardized processing and quality control protocols (218).
Long-Term Safety and Degradation	Unpredictable degradation rates or toxic byproducts (e.g., acidic degradation of PLGA) can harm tissue.	Development of tunable, bioresorbable polymers with safe byproducts (219).
Distinction Between ECM Types	Decellularized ECM and ECM-inspired synthetics are often conflated, obscuring their unique properties.	Clearer classification and reporting standards in research (220).
Limited Mechanistic Insight	Many studies report outcomes without elucidating the underlying cell-ECM signaling mechanisms.	Advanced imaging, omics, and mechanobiology studies (221).
Regulatory and Translational Barriers	Complex compositions and variability complicate regulatory approval for clinical use.	Collaboration with regulatory agencies; development of standards (222).

Future research directions in ECM biomaterials should also emphasize the exploration of interactions between these materials and host tissues at the molecular level. Identifying how various ECM compositions influence cellular responses will produce crucial insights for optimizing scaffold design tailored for specific applications (223). Additionally, employing advanced imaging techniques and *in vivo* models will develop a deeper comprehension of ECM remodeling processes post-implantation and their effects on long-term tissue regeneration (224). Innovative research methodologies can address these challenges, advancing the development of more effective ECM-inspired biomaterials to significantly improve patient outcomes in regenerative medicine.

## 12 Conclusion

ECM-inspired biomaterials have significantly advanced tissue repair and regeneration by mimicking native ECM's biochemical and biophysical properties, promoting cell migration, proliferation, and differentiation, and improving healing. However, clinical translation faces scientific and translational challenges.

A key scientific limitation is the incomplete understanding of how specific ECM components and their spatial arrangements collectively regulate cell behavior. While molecules like collagen, fibronectin, and laminin influence cell adhesion, their complex interactions with growth factors and signaling molecules require further elucidation to rationally design biomaterials that faithfully recreate the native ECM microenvironment.

Immunogenicity remains a concern, with residual immunogenic molecules in decellularized ECM or immune reactions to synthetic materials potentially causing chronic inflammation, fibrosis, or graft failure. Research focuses on improving decellularization, developing immunomodulatory biomaterials, and engineering safer degradation profiles. Achieving effective host tissue integration and vascularization are major translational barriers. Synthetic scaffolds often struggle to support sufficient cell infiltration or blood vessel formation. Strategies like incorporating pro-angiogenic factors or creating pre-vascularized constructs are being explored, but robust vascularization in large or complex tissues remains challenging.

Batch-to-batch variability, especially in ECM-derived materials, hinders reproducibility, quality assurance, and regulatory approval. Standardizing sourcing, processing, and characterization is crucial. Furthermore, the distinction between decellularized ECM scaffolds and ECM-inspired synthetic materials needs clarification for accurate interpretation and tailored therapeutic strategies. Long-term safety and efficacy data are limited, necessitating longitudinal investigations and well-designed clinical trials to evaluate durability, degradation, and biological integration *in vivo*.

Overcoming these hurdles requires interdisciplinary collaboration, integrating advanced biomaterial engineering, high-throughput screening, and systems biology. This will accelerate the development of safe, effective, and innovative ECM-inspired biomaterials.

In conclusion, ECM-inspired biomaterials offer a transformative approach to regenerative therapies. Continued interdisciplinary research is essential to overcome current challenges and engineer intelligent, adaptable, and biocompatible systems for personalized and highly effective regenerative solutions.
