# Supplementary figures and images for: Biomaterials in tissue repair and regeneration: key insights from extracellular matrix biology
Source: Front Med Technol. 2025 Aug 15;7:1565810. doi: 10.3389/fmedt.2025.1565810 (PMC12394198; doi:10.3389/fmedt.2025.1565810)

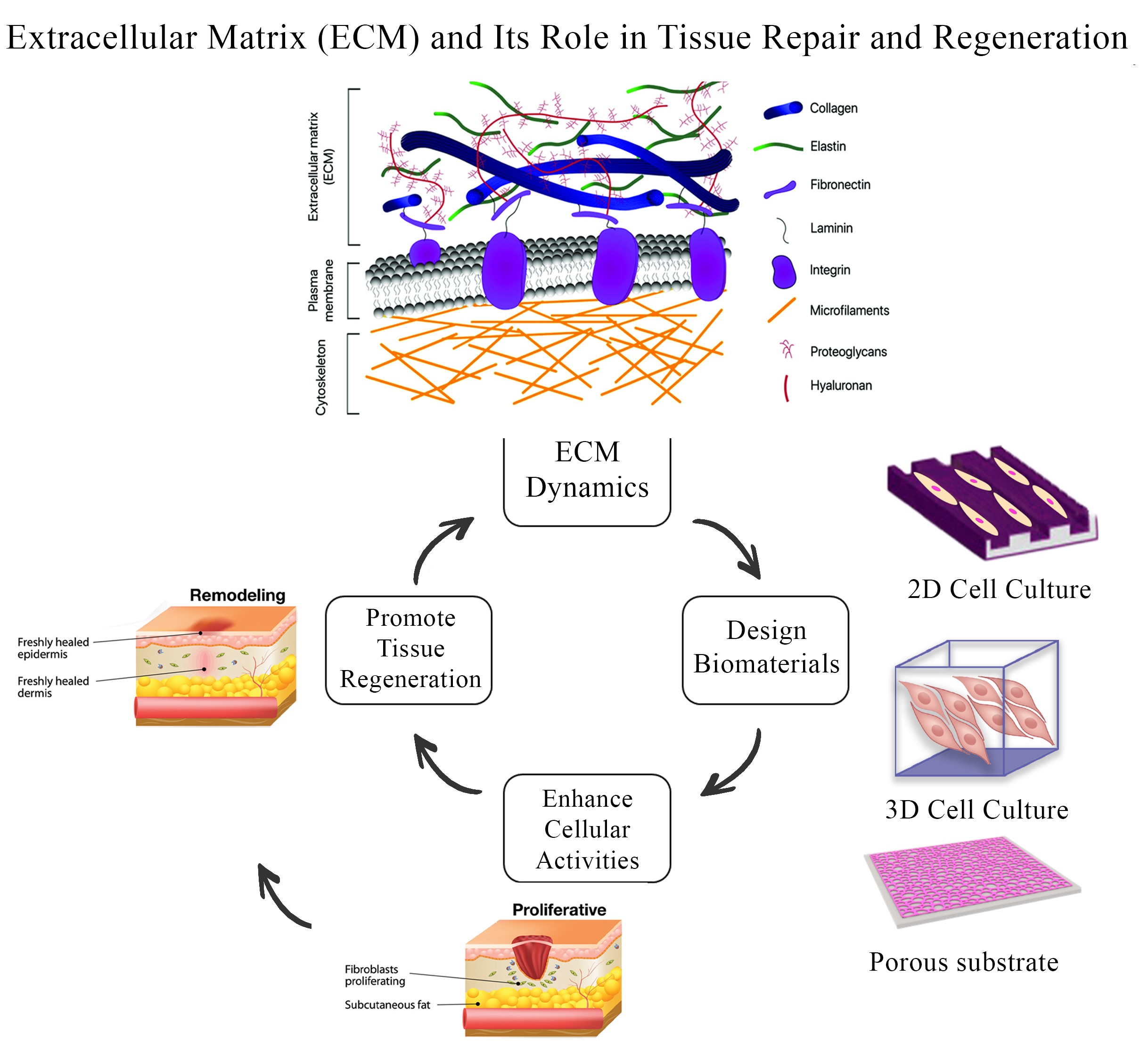

Supplement: Supplementary file 1 [file Image1.jpeg]
